# Editorial Note: TNFα Cooperates with IFN-γ to Repress Bcl-xL Expression to Sensitize Metastatic Colon Carcinoma Cells to TRAIL-mediated Apoptosis

**DOI:** 10.1371/journal.pone.0356584

**Published:** 2026-08-20

**Authors:** The PLOS One Editors

The *PLOS One* Editors issue this Editorial Note to inform readers that since [1] was published, the following concerns have been raised about references cited in the article’s Introduction, Results, and Discussion sections:

- References 22 and 42 have been retracted.
- References 14 and 53 received Expressions of Concern.
- References 17, 20, and 38 received Editor’s Notes.
- Concerns have been raised on PubPeer about results reported in References 15, 18, 37, 52, 56, and 57.

PLOS concluded that the references of concern are not critical in supporting the research reported in [1]. The citing statements report background information and in most cases are supported by additional references.
